# Gastric Mucinous Cancer Histology: Clinicopathological Characteristics and Prognostic Value

**DOI:** 10.1155/2016/8947505

**Published:** 2015-12-29

**Authors:** Chen Jian-Hui, Cai Shi-Rong, Wu Hui, Xu Jian-bo, Wu Kai-Ming, Chen Si-le, Yu-Long He

**Affiliations:** ^1^Division of Gastrointestinal Surgery, The First Affiliated Hospital, Sun Yat-sen University, Guangzhou 510080, China; ^2^Gastric Cancer Center, Sun Yat-sen University, Guangzhou 510080, China

## Abstract

MC tended toward worse tumor biological behavior and long-term survival outcome compared to WMDC. Moreover, MC also showed worse clinicopathological features and survival outcome in some selected patients. For these reasons, MC should be deemed as a special histological type of gastric cancer with worse clinicopathological features and survival outcome.

## 1. Introduction

The adenocarcinoma from several organs can secret the mucinous-like substance, including the gastrointestinal track. The incidence of colorectal mucinous cancer (MC) is higher than that of gastric MC. According to the literature, the colorectal MC accounts for 7.0% to 14.8% [1–3] in all histological types of colorectal cancer, and it indicates larger tumor size, deeper invasion, and poorer survival outcome. Moreover, the MC is a rare histological type in gastric carcinoma, occupying from 3.3% to 7.1% of total gastric cancer patients [4–6]. The pathological manifestation of this histological type is defined by the World Health Organization (WHO) as the mucus aggregates in the tumor stroma and forms mucinous pools, occupying the majority of the whole view in the microscope (more than 50%). The mucinous pool locating intracellularly and small amount of extracellular mucus aggregation are the exclusive criteria of MC.

The clinical characteristics and prognosis of MC are still controversial because of the small sample size of MC cases. Some believed that the MC patients had worse clinical parameters and survival prognosis [6, 7]; some [8] insisted that there were no distribution differences of clinical characteristics and survival difference between MC and nonmucinous cancer (NMC) for gastric cancer. It is worth noting that in these articles the comparison object of MC was the NMC which included both well and moderately differentiated cancer (WMDC) and poor differentiated cancer (PDC) [9]. The different compositions of NMC may lead to the controversial results in the literature. Aiming at finding out the exactly clinical significance and survival outcome of this rare histological type, we compared MC to WMDC and PDC, respectively.

## 2. Materials and Methods

From June 1995 to December 2006, there were consecutive 996 primary gastric adenocarcinoma patients who had undergone palliative or curative gastrectomy with completed data at the Department of Gastrointestinal and Pancreatic Surgery, The First Affiliated Hospital of Sun Yat-Sen University. The uncommon histological types were excluded, such as adenosquamous carcinomas, hepatoid carcinomas, gastric carcinoma with lymphoid stroma, and gastric carcinoma with gastrointestinal stromal tumors.

We adopted the retrospective analysis. The clinical parameters were investigated, such as age, gender, tumor diameter, tumor location, Borrmann types, tumor invasion, lymph node metastasis, distant metastasis, peritoneal dissemination, hepatic metastasis, TNM stage, and operative curability. The operation was deemed as curative when the tumor specimen and regional lymph nodes were completely resected and the resection margin was negative in histological examination. The distant metastasis included peritoneal dissemination, hepatic metastasis, and nonregional lymph node metastasis. The pathological findings were carried out by two special pathologists independently and further confirmed by an experienced pathological expert to make a final diagnosis. The classifications of tumor invasion, lymph node metastasis, and distant metastasis were according to the 7th edition of UICC/AJCC TNM stage. The definitions of histological type for gastric cancer followed the criteria of the WHO classification and were revealed previously. In our study, there were 68 gastric MC patients (accounting for 6.8%), 329 WMDC cases (accounting for 33.0%), and 599 PDC ones (accounting for 60.2%).

All the patients received follow-up programs that followed the concurrent NCCN/AJCC guideline, including body examination, laboratory examination, check X-ray, and abdominal CT scan/abdominal ultrasound and gastroscopy. The follow-up protocol was every 3 months for the postoperative two years, every 4 months for the next 1 year, every 6 months for the next 2 years, and after 5 years every 12 months until death. The latest follow-up date was December 2013. The follow-up period for all the patients was more than 5 years. 4.4% (44/996) patients were lost during follow-up survey.

Chi-square test was used to compare the distribution differences of individual variables between groups. Survival curve was conducted using the Kaplan-Meier method and the survival differences were compared using the log-rank test. Cox proportional hazards regression model and the forward: LR procedure were used for univariate and multivariate analysis. Only the statistically significant prognostic factors in the univariate analysis were further used for multivariate analysis. The accepted level of significance was *P* < 0.05. The statistical package used in this study was the Statistical Package for Social Sciences (SPSS 18.0, Chicago, IL, USA).

## 3. Results

### 3.1. Clinicopathological Parameters Comparison between MC and WGMDC and PDC Patients

The clinicopathological features of MGC, WMDC, and PDC patients were compared (Tables 1 and 2). There was no significant difference of the average age between MC groups and WMDC groups (59.2 years old and 60.8 years old, resp.) and no significant difference in the distribution of the elder proportion was found too. Moreover, the average age of MC patients was higher than that of PDC patients (54.1 years old) and MC group had statistically larger proportion (50.0%) of the elder cases than the PDC ones (36.2%). Moreover, there were no significant differences in the distribution of the gender, tumor location, and Borrmann type among MC group, WMDC group, and PDC one. The mean tumor diameter of MC, WMDC, and PDC was 6.81 cm, 5.18 cm, and 6.31 cm, respectively, and we found more MC patients with larger tumor size (>5 cm) than WMDC cases. From Table 1 we found a significantly worse clinicopathological features of tumor invasion, lymph node involvement, peritoneal seeding, TNM stage in MC patients than in WMDC cases. Moreover, MC patients also exhibited a worse tumor biological behavior of tumor invasion and peritoneal dissemination compared to PDC patients and no distributions of lymph node involvement, TNM stage, and liver metastasis were found between MC group and PDC group. The radical resection rate of different histological types for gastric cancer in the descending sequence is as follows: WMDC (77.8%), PDC (70.3%), and MC (63.2%). A statistical difference of radical resection rate between MC patients and WMDC ones was observed.

### 3.2. Survival Analysis

#### 3.2.1. Survival Comparison between Gastric MC and WMDC Patients

**Figure 1:**
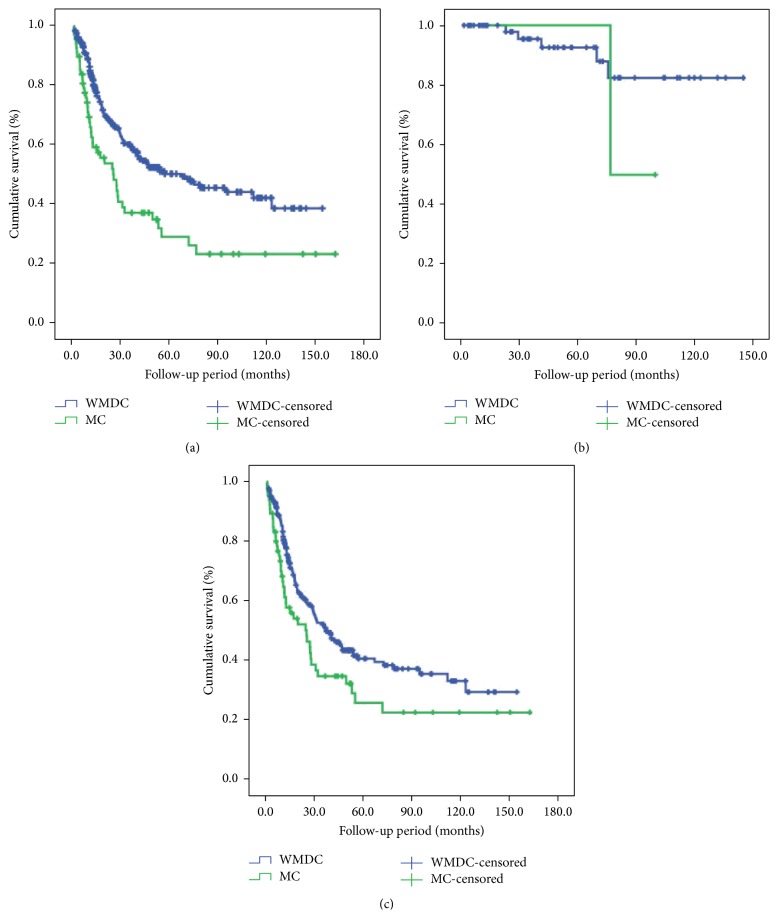
Survival comparison between MC and WMDC patients. (a) Total patients. (b) Patients in the early stage. (c) Patients in the advanced stage.

The median survival time (MST) of MC and WMDC patients was 26.7 months and 67.4 months, respectively. Using Kaplan-Meier analysis, there were statistical differences between MC patients and WMDC patients (*χ* ^2^ = 12.61, *P* = 0.004). However, there was no survival difference between MC and WMDC patients with early stage, and the patients with advanced MC had worse prognostic outcome than the ones with advanced WMDC. The data was shown in Table 3 and Figure 1.

#### 3.2.2. Survival Comparison between MC and PDC Patients

**Figure 2:**
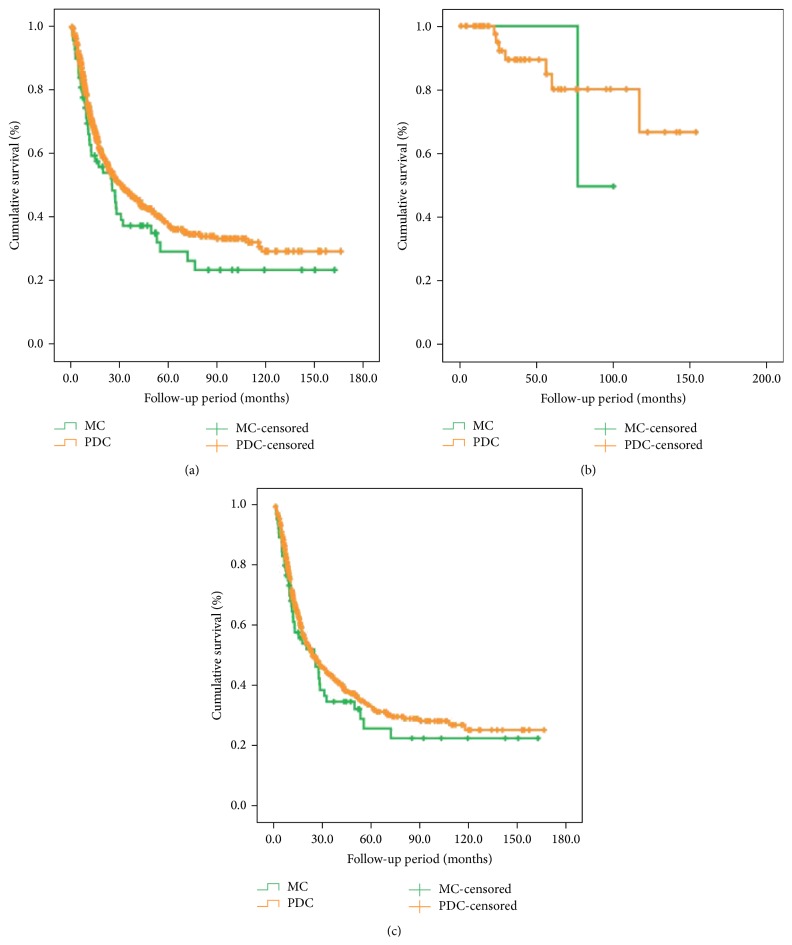
Survival comparison between MC and PDC patients. (a) Total patients. (b) Patients in the early stage. (c) Patients in the advanced stage.

*(1) Overall Survival Comparison*. We found that the long-term survival outcome of PDC patients was better than the one of MC cases, although the survival difference had no statistical significance (*χ* ^2^ = 2.020, *P* = 0.155). Moreover, no survival differences were found between PDC and MC patients no matter in the early stage or advanced stage. The data was shown in Table 4 and Figure 2.

**Figure 3:**
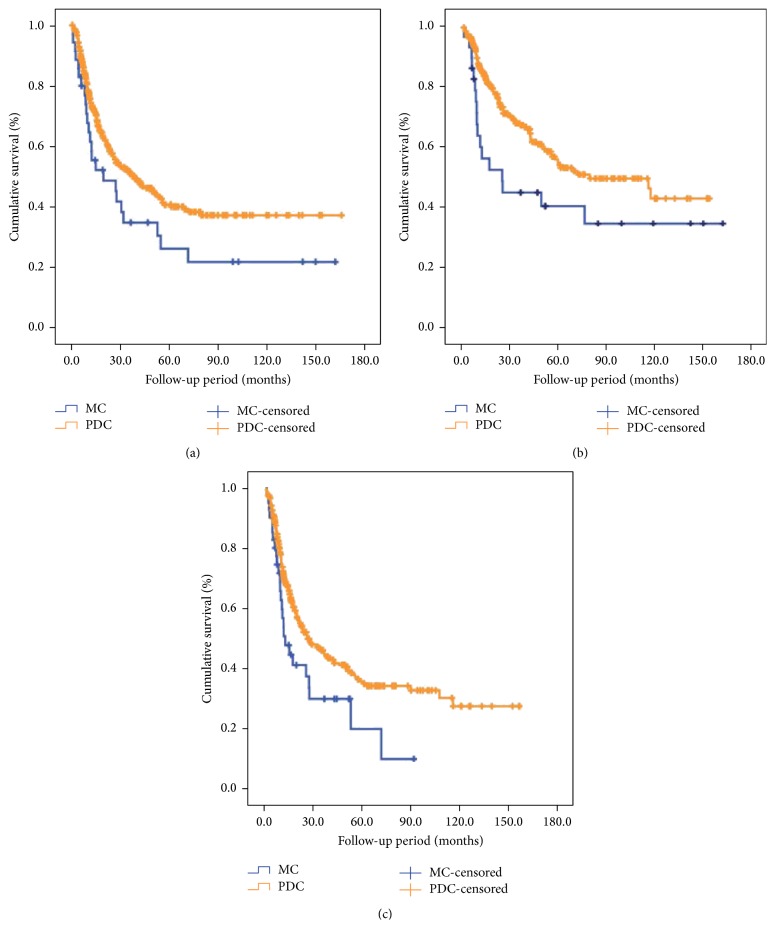
Subgroup survival comparison between MC and PDC patients. (a) Patients with ages ≦ 60 years (*P* = 0.046). (b) Patients with tumor diameter ≦ 5 cm (*P* = 0.029). (c) Patients with Borrmann type III (*P* = 0.023).

*(2) Subgroup Survival Comparison*. We further compared the survival differences between MC patients and PDC ones by stratified analysis. The results in Figure 3 indicated that only when patients are with age ≤ 60 years, tumor diameter ≤ 5 cm, and Borrmann type III, did MC group show a worse survival outcome than PDC groups (Table 5). Subgroup analysis for the parameters of gender, tumor location, depth of invasion, lymph node metastasis, peritoneal metastasis, liver metastasis, TNM stage, and radical resection did not affect the survival outcome between two groups.

### 3.3. Cox Regression Analysis

The histological analysis in our study was divided into three parts: WMDC group, MC group, and PDC group. As shown in Table 6, univariate regression analysis shows that the elder age, Borrmann type, histological types, tumor diameter, depth of invasion, lymph node metastasis, distant metastasis, TNM stage, radical resection, and chemotherapy affected the overall survival prognosis in our study. Only the significantly statistical prognostic factors in the univariate analysis were used for further multivariate analysis. And the multivariate Cox regression analysis indicated that age, tumor diameter, lymph node metastasis, TNM classification, adjuvant chemotherapy, and radical dissection were the independent prognostic factors. However, the histological factor was not the independent prognostic factor for gastric cancer in our study.

## 4. Discussion

There were many classifications of histological types for gastric cancer, such as Lauren classification [10], Ming classification [11], and WHO classification. Nowadays, WHO classification is widely used worldwide. The WHO histological classification for gastric cancer can divide into well differentiation types (well differentiated and moderately differentiated) and poor differentiation types (poorly differentiated and undifferentiated) [12]. The well differentiation types included papillary and well differentiated and moderately differentiated cancer, and the poor differentiation types included poor differentiated and mucinous cancer and signet ring cell carcinoma and undifferentiated carcinoma. MC, a rare kind of poor differentiation histological type, had abundant mucus in the tumor issue, with nest-like or mass shape generated by the tumor cell accumulating in the cancer nests. However, controversy still existed on the clinicopathological characteristics and prognostic factors.

In our study, we found that MC had a tendency to have larger tumor size, deeper gastric wall invasion, more frequent lymph node involvement, more advanced tumor stage, more peritoneal dissemination, and less curative rate than WMDC. Similar findings were reported in the previous studies [5–7] when MGC was compared to NMC. However, there were little studies to compare MC to PDC. Some authors [4, 13] just compared the clinicopathological characteristics of MGC to a small part of PDC, gastric signet ring cell cancer (GSRCC). In our study, we found out that although the clinicopathological features of MC were worse than those of PDC, the differences in the distribution of the clinicopathological features between MGC and PDC were smaller. There were distribution differences only in the cases with senile ones, gastric wall invasion, and peritoneal dissemination. Hence the opposite conclusions made by Kawamura et al. [14] and Adachi et al. [8] may be due to the different proportion of PDC in the NMC patients (Kawamura: 53.2% and Adachi: 41%).

Up to the present, most of the authors insisted that MC represented a worse tumor biological behaviors and had deeper invasion, more lymph node involvement, more advanced tumor stage, and low radical resection rate, which led to a poorer survival outcome than NMC [5]. Nevertheless, some revealed that there was no survival difference between MC and NMC for the patients with the same stage [6, 15]. In order to explore the exact survival outcome of MC, our study distinguished the survival differences between MC, WMDC, and PDC. The survival times in the descending order were listed as follows: WMDC (50.9%), PDC (38.4%), and MC (29.4%). A significantly statistical difference was found between WMDC and MC and there was no survival difference between MC and PDC. Interestingly, the patients with age ≤ 60 years, tumor diameter ≤ 5 cm, and Borrmann type III in the MC group showed a worse overall survival outcome than those in the PDC group. Our result may give help to explain and distinguish survival results. We found that a larger proportion of PDC in the NMC may lead to a smaller survival difference. Kawamura et al. [14] showed a worse survival outcome of MC than of NMC and PDC accounted for 45.1% in NMC, while Park held an opposite view and PDC accounted for 50.1% in NMC. This comparison showed that, with the smaller proportion of PDC, MC showed a poorer outcome compared with NMC.

Similar to most of the authors [7, 16, 17], the mucinous histological type itself was not an independent prognostic factor in Cox proportional hazard model in our study, but age, tumor diameter, depth of invasion, lymph node metastasis, TNM stage, adjuvant chemotherapy, and radical resection were independent prognostic factors in our study. It was possible that most of the gastric patients in our study were detected in the advanced stage at diagnosis and the mucinous histological type had little prognosis significance.

In conclusion, MC tended toward worse tumor biological behavior and long-term survival outcome compared to WMDC. Moreover, MC also showed worse clinicopathological features (more senile people, advanced tumor invasion, and frequent peritoneal metastasis). No survival difference was found between MC and PDC. Only MC patients with age ≤ 60 years, tumor diameter ≤ 5 cm, and Borrmann type III, showed a worse survival outcome than did PDC groups. For these reasons, MC should be deemed as a special histological type of gastric cancer.

## Figures and Tables

**Table 1:** Comparison of clinicopathological features between MC and WMDC patients for gastric cancer.

	MC (68 cases)	WMDC (329 cases)	*χ* ^2^ value	*P* value
Age			0.571	0.450
≤60 years	34 (50.0%)	148 (45.0%)		
>60 years	34 (50.0%)	181 (55.0%)		
Gender			3.513	0.061
Male	44 (64.7%)	249 (75.5%)		
Female	24 (35.3%)	80 (24.3%)		
Tumor diameter			8.867	0.003
≤5 cm	28 (41.2%)	200 (60.8%)		
>5 cm	40 (58.8%)	129 (39.2%)		
Tumor location			1.470	0.479
Upper 1/3	25 (36.8%)	132 (40.1%)		
Middle 1/3	7 (10.3%)	47 (14.3%)		
Lower 1/3	36 (52.9%)	150 (45.6%)		
Borrmann type			7.399	0.060
Borrmann I	8 (11.8%)	30 (9.1%)		
Borrmann II	12 (17.6%)	113 (34.3%)		
Borrmann III	41 (60.3%)	162 (49.2%)		
Borrmann IV	7 (10.3%)	24 (7.3%)		
Depth of invasion			22.274	<0.001
T_1_	2 (2.9%)	60 (18.2%)		
T_2_	7 (10.3%)	77 (23.4%)		
T_3_	33 (48.5%)	125 (38.0%)		
T_4_	26 (38.2%)	67 (20.4%)		
Lymph node metastasis			10.130	0.017
N_0_	13 (19.1%)	121 (36.8%)		
N_1_	15 (22.1%)	76 (23.1%)		
N_2_	23 (33.8%)	82 (24.9%)		
N_3_	17 (25.0%)	50 (15.2%)		
TNM stage			10.827	0.013
I	6 (8.8%)	82 (24.9%)		
II	11 (16.2%)	58 (17.6%)		
III	28 (41.2%)	119 (36.2%)		
IV	23 (33.8%)	70 (21.3%)		
Peritoneal dissemination			11.849	0.001
P (+)	20 (29.4%)	42 (12.8%)		
P (−)	48 (70.6%)	287 (87.2%)		
Hepatic metastasis			0.386	0.535
H (+)	3 (4.4%)	21 (6.4%)		
H (−)	65 (95.6%)	308 (93.6%)		
Curability			6.440	0.011
Curative	43 (63.2%)	256 (77.8%)		
Noncurative	25 (36.8%)	73 (22.2%)		

**Table 2:** Comparison of clinicopathological features between MC and PDC patients for gastric cancer.

	MC (68 cases)	PDC (599 cases)	*χ* ^2^ value	*P* value
Age			4.936	0.026
≤60 years	34 (50.0%)	382 (63.8%)		
>60 years	34 (50.0%)	217 (36.2%)		
Gender			0.032	0.858
Male	44 (64.7%)	381 (63.6%)		
Female	24 (35.3%)	218 (36.4%)		
Tumor diameter			2.318	0.128
≤5 cm	28 (41.2%)	305 (50.9%)		
>5 cm	40 (58.8%)	294 (49.1%)		
Tumor location			3.271	0.195
Upper 1/3	25 (36.8%)	185 (30.9%)		
Middle 1/3	7 (10.3%)	115 (19.2%)		
Lower 1/3	36 (52.9%)	299 (49.9%)		
Borrmann type			5.997	0.112
Borrmann I	8 (11.8%)	52 (8.7%)		
Borrmann II	12 (17.6%)	191 (31.9%)		
Borrmann III	41 (60.3%)	301 (50.3%)		
Borrmann IV	7 (10.3%)	55 (9.2%)		
Depth of invasion			7.864	0.049
T_1_	2 (2.9%)	59 (9.8%)		
T_2_	7 (10.3%)	101 (16.9%)		
T_3_	33 (48.5%)	280 (46.7%)		
T_4_	26 (38.2%)	159 (26.5%)		
Lymph node metastasis			5.435	0.143
N_0_	13 (19.1%)	161 (26.9%)		
N_1_	15 (22.1%)	172 (28.7%)		
N_2_	23 (33.8%)	142 (23.7%)		
N_3_	17 (25.0%)	124 (20.7%)		
TNM stage			3.514	0.319
I	6 (8.8%)	95 (15.8%)		
II	11 (16.2%)	115 (19.2%)		
III	28 (41.2%)	228 (38.1%)		
IV	23 (33.8%)	161 (26.9%)		
Peritoneal dissemination			5.858	0.016
P (+)	20 (29.4%)	104 (17.4%)		
P (−)	48 (70.6%)	495 (82.6%)		
Hepatic metastasis			0.001	0.971
H (+)	3 (4.4%)	27 (4.5%)		
H (−)	65 (95.6%)	572 (95.5%)		
Curability			1.433	0.231
Curative	43 (63.2%)	421 (70.3%)		
Noncurative	25 (36.8%)	178 (29.7%)		

**Table 3:** Survival comparisons between MC and WMDC patients for gastric cancer.

	WMDC	MC	*χ* ^2^ value	*P* value
Total			12.61	0.004
1-year OS rate	83.4%	64.3%		
3-year OS rate	60.5%	37.1%		
5-year OS rate	50.9%	29.4%		
Early stage			0.676	0.411
1-year OS rate	100.0%	100.0%		
3-year OS rate	95.5%	100.0%		
5-year OS rate	92.5%	100.0%		
Advanced stage			5.741	0.017
1-year OS rate	79.8%	63.2%		
3-year OS rate	52.3%	34.8%		
5-year OS rate	41.6%	26.5%		

OS: overall survival.

**Table 4:** Survival comparisons between MC and PDC patients for gastric cancer.

	PDC	MC	*χ* ^2^ value	*P* value
Total			2.020	0.155
1-year OS rate	72.2%	64.3%		
3-year OS rate	47.9%	37.1%		
5-year OS rate	38.4%	29.4%		
Early stage			0.402	0.526
1-year OS rate	100.0%	100.0%		
3-year OS rate	89.5%	100.0%		
5-year OS rate	85.0%	100.0%		
Advanced stage			0.837	0.360
1-year OS rate	69.2%	63.2%		
3-year OS rate	43.6%	34.8%		
5-year OS rate	33.7%	26.5%		

OS: overall survival.

**Table 5:** Significant subgroup survival comparisons between MGC and PDC patients.

	MGC	PDC	*χ* ^2^ value	*P* value
Age ≤ 60 years			3.968	0.046
1-year OS rate	64.7%	75.2%		
3-year OS rate	34.7%	51.6%		
5-year OS rate	26.0%	40.7%		
Tumor diameter ≤ 5 cm			4.796	0.029
1-year OS rate	60.7%	85.9%		
3-year OS rate	45.5%	68.3%		
5-year OS rate	40.5%	56.6%		
Borrmann III type			5.152	0.023
1-year OS rate	55.3%	70.8%		
3-year OS rate	30.5%	46.4%		
5-year OS rate	22.8%	36.6%		

**Table 6:** Cox regression analysis results for gastric cancer.

Factors	Univariate regression analysis			Multivariate regression analysis		
	RR	95% CI	*P* value	RR	95% CI	*P* value
Elder age	1.222	1.022–1.461	0.028	1.341	1.118–1.610	0.002
Gender	0.689	—	0.407			
Tumor diameter	2.999	2.487–3.616	<0.001	1.560	1.283–1.896	<0.001
Histological type	1.144	1.051–1.246	0.002			
Tumor location	0.199	—	0.655			
Borrmann type	2.068	1.814–2.358	<0.001			
Depth of invasion	2.891	2.519–3.318	<0.001			
Lymph nodes metastasis	2.899	2.535–3.316	<0.001	1.719	1.447–2.041	<0.001
TNM stage	1.986	1.851–2.130	<0.001	1.374	1.242–1.520	<0.001
Adjuvant chemotherapy	0.740	0.601–0.910	0.004	0.752	0.607–0.931	0.009
Radical resection	6.705	5.530–8.129	<0.001	2.453	1.945–3.095	<0.001
